# Olive Production Threatened by a Resurgent Pest *Liothrips oleae* (Costa, 1857) (Thysanoptera: Phlaeothripidae) in Southern Italy

**DOI:** 10.3390/insects11120887

**Published:** 2020-12-16

**Authors:** Gregorio Vono, Carmelo Peter Bonsignore, Gregorio Gullo, Rita Marullo

**Affiliations:** 1 Dipartimento di Agraria, Università degli Studi Mediterranea di Reggio Calabria, 89122 Reggio Calabria, Italy; ggullo@unirc.it (G.G.); rmarullo@unirc.it (R.M.); 2 Laboratorio di Entomologia ed Ecologia Applicata, Dipartimento Patrimonio Architettura Urbanistica, Università degli Studi Mediterranea di Reggio Calabria, 89124 Reggio Calabria, Italy; cbonsignore@unirc.it

**Keywords:** *Liothrips oleae*, *Oleae europea*, olive pest, phytosanitary management, molecular characterization

## Abstract

**Simple Summary:**

*Liothrips oleae* (Costa, 1857) (Thysanoptera: Phlaeothripidae) is widespread in the Mediterranean area, and in all regions, it is reputed to be a secondary pest in olive crops, mainly associated with damage to leaves and secondarily with damage to drupes, for which this thrips pest has a marginal impact on olive production. Taking into account the increase in the frequency of extreme damage by this species in Southern Italy in the last decade, this research aimed to elucidate the real impact (i.e., damage to leaves and drupes) by feeding thrips’ activity in olive orchards. Our results revealed that the impact of thrips was significant in all monitored olive orchards and the estimated damage level on drupes and leaves was higher in organic olive than in integrated olive management. A detailed morphological description of the Italian specimens and their molecular characterization are also provided.

**Abstract:**

This study investigated a resurgence of *Liothrips oleae* Costa (Thysanoptera: Phlaeothripidae), an insect pest of olive crops, in a focal Southern Italian olive-producing area (Calabria Region). The young and adult olive thrips feed on the leaves and fruits of wild and cultivated olive trees, producing distortions, necrosis, and premature dropping of fruit. In our study, organic and integrated olive groves were compared for two years in order to establish the relationship between leaf and fruit damage among olive groves managed under different phytosanitary conditions. Sampling techniques were used in order to collect and count leaves and fruits (on plants and dropped premature drupes) presenting symptoms of thrips’ feeding activity. The impact of the thrips was significant in all orchards, and the estimated damage level on drupes and leaves was higher in organic olive management in each year. A morphological description of the adult females of the species is provided, and the first molecular characterization of the Calabrian olive thrips population was performed by using three different genetic regions (cytochrome c oxidase subunit I (COI), 28S ribosomal subunit (28S), and internal transcribed spacer 2 (ITS2)).

## 1. Introduction

*Liothrips oleae* (Costa, 1857) is normally viewed as a secondary pest in many olive-growing areas [1,2], and it has rarely been considered harmful to the vegetative (leaves and shoots) and reproductive structures (drupes) of plants. The geographical distribution of the species comprises the countries of the Mediterranean region, including those of both the European coast and the Northern African side, where olive cultivation is largely developed and plant species of the genus *Olea* are native. Moreover, *L. oleae* has been also recorded in Poland [3] and Yemen [4].

Reports of severe thrips infestations of specialized olive crops in the Calabria Region of Southern Italy [5], from the late spring to mid-summer of 2017 and in the following years, always in the same seasonal period, have been recorded. Samples of olive plant parts (sprouts and drupes with evident symptoms of thrips attacks) as well as specimens (mainly adults) have been sent to the Entomology Section of the Agriculture Department at the Mediterranean University of Reggio Calabria. This has led to the identification of the responsible species, *Liothrips oleae* (Costa, 1857), a phlaeothripid belonging to the order of Thysanoptera. *L. oleae* has been found to exclusively affect plants of the *Olea* genus. In general, some species of the genus *Liothrips* have been studied for their potential role in biological control, for example, *Liothrips tractabilis* [6], which has been recorded in a control program of invasive herbs [7], and for the role they play in damaging the vegetative structure and modifying the architecture of plants [8,9].

Priesner [10] included the species in the genus *Liothrips*, whereas Uzel [11] and Mound [12] recognized the synonym of *Leurothrips linearis* that Bagnall described in 1908 [13] with *L. oleae*. The genus *Liothrips* is among the largest in the Thysanoptera order, including approximately 230 listed species when also considering those from the synonymic genus *Rhynchothrips*. In this study, the identification of the genus *Liothrips* was treated in the sense of Stannard [14] to include a wide range of dark-bodied species with one sense cone on antennal segment III and three on antennal segment IV, the pronotum with five pairs of major setae, the prosternal basantra absent, and most of the body setae long and dark.

**Figure 1:**
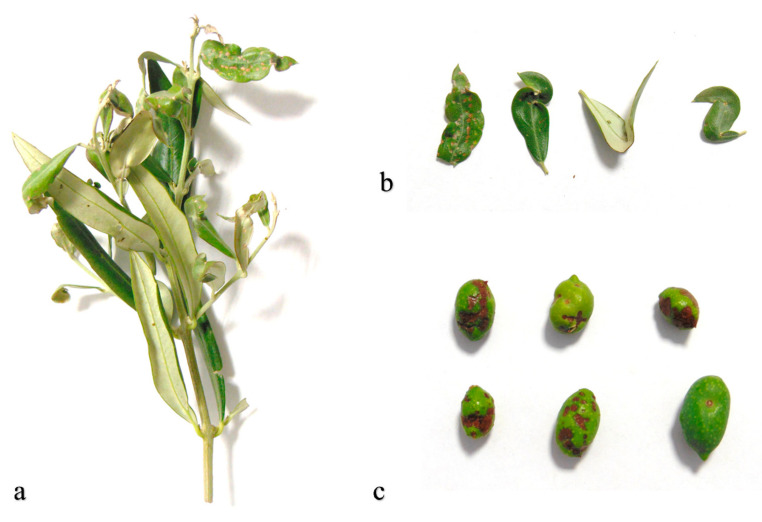
(**a**) Damage to new shoots; (**b**) damage to leaves; (**c**) damage to olive fruits.

The life cycle of the species coincides with the beginning of spring. Usually, in April, after mating, a large number of males appear, while females lay during May. One female lays approximately 200 eggs, which will hatch after approximately 15 days. The young nymphs live gregariously on tender shoots and leaves. The appearance of adults occurs after 18–20 days. Generally, the species has three annual generations. The first adult generation appears early in July, and a second larvae generation in the middle of the month. Immature stages are scarce from mid-August to the end of September. Adult females lay their eggs into bark plants or on the underside of leaves near the main rib. Adults hibernate in the galleries of Scolytids and in other sheltered places on the trees from November [15,16,17]. *L. oleae* has been observed only on *Olea* spp., although some adults may have been taken from related plant species inside the olive groves. Symptoms of heavy infestations on olive drupes and leaves can be seen from the end of spring to the beginning of winter (Figure 1a–c).

*Liothrips oleae* is amply diffused, and natural antagonists can control its possible infestations [15]. Among its natural enemies, Silvestri [15] reported an unidentified Cecidomyiid, the Eulophid *Tetrastichus gentilei* (del Guercio), which sometimes parasitizes up to 75% of the larvae and completes its life cycle in approximately 20 days, as well as an Anthocorid (*Ectemnus reduvinus*, H.-S.), including its nymphs and, to a lesser extent, its adults, which destroy large numbers of thrips [15].

In relation to the new phytoiatric emergency in Southern Italian olive crops, investigations have been carried out to quantify the possible factors causing this secondary pest to act as a key parasitic insect. The aim of this study was to determine the damage caused by thrips on different parts of the olive plant and to assess its damage according to evaluable specific symptoms that characterize the feeding activity of herbivores. In addition, we used a comparative method to evaluate how phytosanitary management (organic vs. integrated) could affect damage to olive drupes by *Liothrips oleae*. In consideration of the difficulties in recognizing the thrips species in this study, the morphological description of the adult female specimens collected was based on the main characters, and their molecular characterization was performed by using three different genetic regions.

## 2. Materials and Methods

### 2.1. Study Site

This study involved four olive groves located in the territory of Catanzaro on the Ionian coast, where Carolea cv. represents the most commonly grown olive variety. The olive groves are located at altitudes between 131 and 460 m. The direct distance between the olive fields is more than 600 m, and they are grown with two different phytosanitary management systems (organic and integrated) (Table 1). The olive groves are cultivated in pot form, and their age varies from 40 to 60 years. In integrated management, the main insecticide treatments concern attacks of *Bactrocera oleae* (Diptera, Tephritidae) and, in some years, a few interventions were carried out against *Prays oleae* (Lepidoptera: Praydidae). Phytoiatric interventions in the sampled olive groves (IPM management) include two insecticide treatments with dimethoate during the production season after July and three phytosanitary treatments (pyraclostrobin and cupric products) against fungal attacks.

The climate at the study site is marked by a rainy season from November to March, followed by a dry summer and autumn from June to September. For the olive groves investigated in the years of the experiment, insecticide treatments were limited to two sprayings with dimethoate. For organic olive groves, no intervention with an eco-sustainable product was adopted.

### 2.2. Sampling of Olive Fruits and Olive Shoots

In each olive grove, a field monitoring plan was designed to be carried out weekly. In relation to the possible herbivore damage to drupes and leaves, in July (2018 and 2019), four plants homogeneous in age and productivity were selected from each field, with a total of 400 drupes and 100 sprouts of a minimum of 15 cm in length (100 drupes and 50 shoots per plant) sampled. The samples were collected from the different sectors of exposure (northeast (NE); southeast (SE); southwest (SW); northwest (NW), and the middle of the plant). All drupes were examined under a stereoscopic microscope (Olympus SZX 9), and the leaves with symptoms of thrips feeding were counted. The evaluation of the damage to the drupes was derived from the number of necrotized stings, considering scale values from 0 to 3 (0 = drupes free of bites from thrips; 1 = drupes with 1–3 bites; 2 = drupes with 4–10 bites; 3 = drupes with over 10 bites; Figure 1c). Moreover, for each drupe, the mean diameter was calculated. The damage, relating the premature dropping of drupes, caused by thrips feeding was determined one time in each study year by selecting at random 4 trees from each olive orchard. To assess damage, we counted the number of prematurely dropped drupes in four iron quadrats (100 cm × 100 cm−1 m^2^). Iron quadrats were placed evenly around the base of each randomly selected tree at ≈180 cm from its base in the four directions of sun exposure (northeast, southeast, southwest, and northwest). The drupes were harvested from each tree’s quadrants every 3 days for three weeks. A drupe was determined to have been dropped by *L. oleae*, rather than simply falling off, if it showed typical symptoms of infestation (see above).

### 2.3. Thrips Sample Collection

The vegetable parts of olive trees that had symptoms caused by pest attack were collected. Then, the samples were sorted on the basis of their morphological characteristics and were inserted individually into an Eppendorf tube containing 98% alcohol. The samples were then inserted into plastic clips with descriptions. The location of sampling was marked using a Global Positioning System (GPS) to obtain coordinates.

### 2.4. Morphological Identification of the Target Thrips Species

The morphological identification of the species was performed using the keys of identification in Mound and Kibby [18], Marullo [19], and ThripsID [20].

The slide preparation of adult specimens of both sexes was based on the recorded methods of Mound and Marullo [21] and Marullo [19]. Voucher specimens were deposited in the Dipartimento di Agraria, Università degli Studi *Mediterranea*, Reggio Calabria, Italy.

### 2.5. Molecular Identification of the Southern Italian L. oleae Adult Specimens

#### DNA Extraction, Amplification, and Sequencing

Samples of adult specimens of *L. oleae* (20 for each olive orchard) were collected from olive trees at monitoring sites and were stored individually in Eppendorf tubes with absolute ethanol at −20 °C. Rather than grinding the specimens, total genomic DNA was extracted using a Chelex–proteinase K-based nondestructive method [22]. DNA extraction was performed on individual insects, incubated in 5 μL of proteinase K (20 mg/mL) and 80 μL of 5% Chelex 100 suspension at 55 °C for 1 h. Proteinase K was then inactivated at 100 °C for 8 min. The supernatant containing the DNA was removed after centrifugation and stored at −20 °C. Three genes were sequenced: the mitochondrial cytochrome c oxidase subunit I (COI), and two nuclear ribosomal regions, namely, the expansion segment D2 of the 28S ribosomal subunit (28S-D2), and the internal transcribed spacer 2 (ITS2).

Polymerase chain reaction (PCR) was used to amplify a fragment of the mitochondrial COI using the primers HCO-2198 forward (5’-TAAACTTCAGGGTGACCAAAAAATCA-3′) and LCO-1490 reverse (5’- GGTCAACAAATCATAAAGATATTGG-3’) [23] and a long fragment of ITS2 and 28S-D2 ribosomal DNA using universal primers ITS2 forward (5’- TGTCAACTGCAGGACACATG -3’) and D2R reverse (5’- TTGGTCCGTGTTTCAAGACGGG -3’) [24].

For COI (~700 bp), ITS2, and 28S-D2 (~1200 bp) fragments, PCR amplification was performed on a Mastercycler^®^ Nexus X2 Series thermocycler using 20 μL reaction volumes, consisting of 1 × Promega PCR buffer (containing MgCl_2_), 0.2 mM of each dNTP, 0.25 μM of each primer, 10 mg/mL bovine serum albumin, 1.5 units Go*Taq G2* DNA polymerase (Promega Italia, Milan, Italy), and 2 μL of DNA template. The thermocycler conditions were as follows: Initial denaturation at 95 °C for 1 min, followed by 40 cycles at 94 °C for 30 s, 48 °C for 90 s, 72 °C for 1 min, and a final extension at 72 °C for 7 min. The thermocycler conditions for ITS2-28S-D2 were as follows: initial denaturation at 93 °C for 5 min, followed by 34 cycles at 93 °C for 15 s, 48 °C for 45 s, 72 °C for 45 s, and a final extension at 72 °C for 7 min. The concentration of the DNA samples was determined by Nanodrop analysis (qualitative and quantitative), and the PCR products were checked on a 1.2% agarose gel stained with GelRED^®^ (Biotium, Fremont, CA, USA), visualized and photographed under UV light. All PCR products produced a single band and were cleaned using the ExoSAP protocol. To confirm the identity of *L. oleae*, Sanger sequencing was performed in both directions through the same primer pairs used for the amplification reactions.

All sequences were aligned via manual trimming in BioEdit version 7.2.5 [25] and were virtually translated into the corresponding amino acid chain to detect frame-shift mutations and stop codons using EMBOSS Transeq [26]. Edited sequences were checked against the GenBank database and BOLD using “BLASTn” [27]. However, no gene sequence for *L. oleae* appeared in the research records (accessed on 30 March 2020). All sequences obtained in this study were submitted to the GenBank database under the accession numbers reported in Table 2. To demonstrate the COI genetic differences between *Liothrips oleae* and other *Liothrips* species (available in genetic database), a phylogenetic tree was developed. Using Partition Finder version 2.1.1 [28], the best-fitted model was identified, and then cluster analysis was carried out using the maximum likelihood (ML) method [29] via MEGA version 7 [30]. Bootstrap analysis was performed based on 1000 resampling. The COI sequence of *Gynaikothrips ficorum* (KX687006) was used as an outgroup (Figure 3).

### 2.6. Statistical Analysis

Data on the damage levels of the drupes and leaves were tested for normality using Kolmogorov–Smirnov (K-S) tests (*p* = 0.05). We used a generalized linear model to assess the damage to drupes and leaves on the plants. The multinomial model (link function: cumulative logit) with damage level intervals of 0–3 was used to compare differences in damage to the drupes in each year. The categorical variables of phytosanitary management and the compass direction of the drupes (*n* = 5; NE, SE, SW, NW, and the center of the plant) were included. The mean diameter of the drupes was added as a continuous variable. For symptomatic leaves, a normal model was used with the total number of leaves used as a scaled weight variable. Finally, comparisons of the damage level of the drupes and the mean number of leaves with symptoms at each sector of exposure were made by calculating partial correlation coefficients, with the total number of leaves held constant. A generalized linear model for counting the data to compare differences in the premature drop of drupes from canopy plants was used.

We used SPSS version 23 [31] for all data analyses and Sigmaplot 13.0 [32] (2018, Systat Software, San Jose, CA, USA) to produce graphs. All data are expressed as untransformed mean values ± standard error (SE).

## 3. Results

### 3.1. Morphological Description of the Southern Italian L. oleae Specimens

The morphological identification of the adult specimens of both sexes taken from the monitored olive trees revealed that they all belonged to the thrips species *Liothrips oleae* (Costa), commonly known as “olive thrips.” It belongs to the sub-order Tubulifera and to the family Phlaeothripidae, and is widespread and common on olive trees.

**Figure 2:**
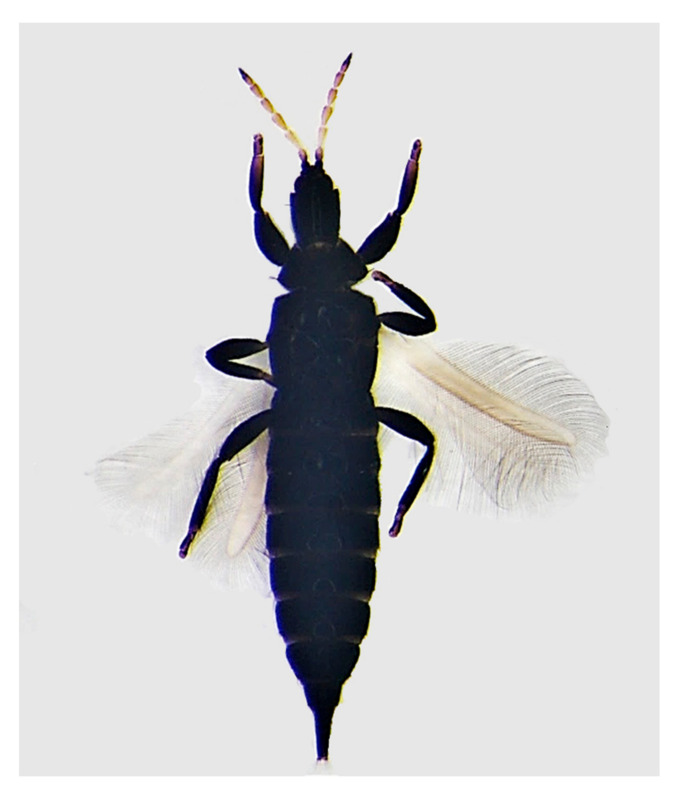
*Liothrips oleae.* Slide-mounted adult female.

Female macropterous (Figure 2): dark brown body, including legs, antennal segments III–VI and basal half of VII yellow, forewings pale but slightly darker distally, major head and pronotum setae dark brown, tergite X setae pale brown. Antennae eight-segmented, segment III with one sense cone, segment IV with three sense cones, all of them being emerged and simple sensoria. Head longer than it is wide, without a pair of stout setae at the basal third of cheeks; maxillary stylets retracted to postocular setae, without a bridge. Postocular setae shorter than the distance of the setal base from the eye, each with a capitate or broadly expanded apex. Pronotum, faintly sculpturated, with five pairs of stout elongated major setae, the posteriors longer than the anteriors; basantra absent. Metanotum with closely spaced longitudinal and anastomosing reticulations. Forewings parallel-sided margins, with more than 20 pairs of duplicated cilia and three stout sub-basal setae. Tergite I with a triangular-shaped pelta, tergites II–VII with two pairs of curved wing-retaining setae each, tergite IX setae capitate, tergite X complete and as long as the width of the head.

Male macropterous: similar to the female; short and thickened setae B2 of tergite IX; sternite VIII without a glandular area; aedeagus with a pair of sclerotized ridges at the apex (similar to hooks when viewed in profile).

### 3.2. Larvae

Larval instars I and II are whitish with red eyes. The dark antennae and legs, the head, and the last abdominal segments have a dark plaque on the sides. The prepupa is orange in color, and all of the dark areas in the body are paler at this stage. The antennae are very short. Two orange nymphal stages follow: their antennae are turned backward along the cheeks and the wing sketches are well evident.

### 3.3. Molecular Analysis

#### 3.3.1. Amplification and Alignment of the COI, ITS2, and 28S Genes

The polymerase chain reaction of *mt*-COI produced fragments of ±620 bp, and, after trimming, the final alignment consisted of 482 bp. The nucleotide composition of these sequences was T(U) = 29.9%, A = 44.6%, C = 13.6%, and G = 11.9%. The average A + T content was high (74.50%), which is in agreement with values for insects in general [23,33]. The nucleotide compositions of two nuclear ribosomal regions sequences were T(U) = 18.4%, A = 19.9%, C = 34.2%, and G = 29.1% for 28S fragments and T(U) = 19.4%, A = 23.1%, C = 27%, and G = 30.6% for ITS2 fragments.

There were no molecular differences among the COI sequences obtained from all thrips samples analyzed. Similar results are evident in the sequences of both regions of rRNA. Alignment of sequences, through the use of BioEdit software, showed no existence of haplotypes in the olive thrips populations analyzed in the present study. In particular, the PCR produced fragments of ±675 bp for the highly conserved 28S genetic region and a ±627 bp aligned matrix of ITS2 sequences.

**Figure 3:**
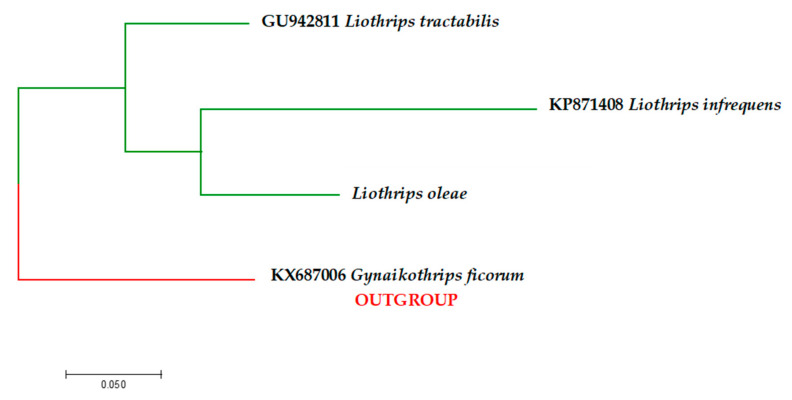
Bootstrap consensus tree generated using the maximum likelihood (ML) method and general time reversible (GTR) G + I (gamma + invariant) model showing the genetic differences and relationship between *Liothrips oleae* obtained by *mt-*COI sequences and all other *Liothrips* species available in GenBank. *Gynaikothrips ficorum* was used as an outgroup. Species name and GenBank accession number are shown in the figure.

A maximum likelihood phylogenetic tree based on the general time-reversible model [34] of mitochondrial genes (*COI*) was constructed using MEGA version 7 software, using the general time reversible (GTR) G + I (gamma + invariant) model, suggested by Partition Finder version 2.1.1 (Figure 3).

#### 3.3.2. Damage Levels of the Drupes and Leaves

**Figure 4:**
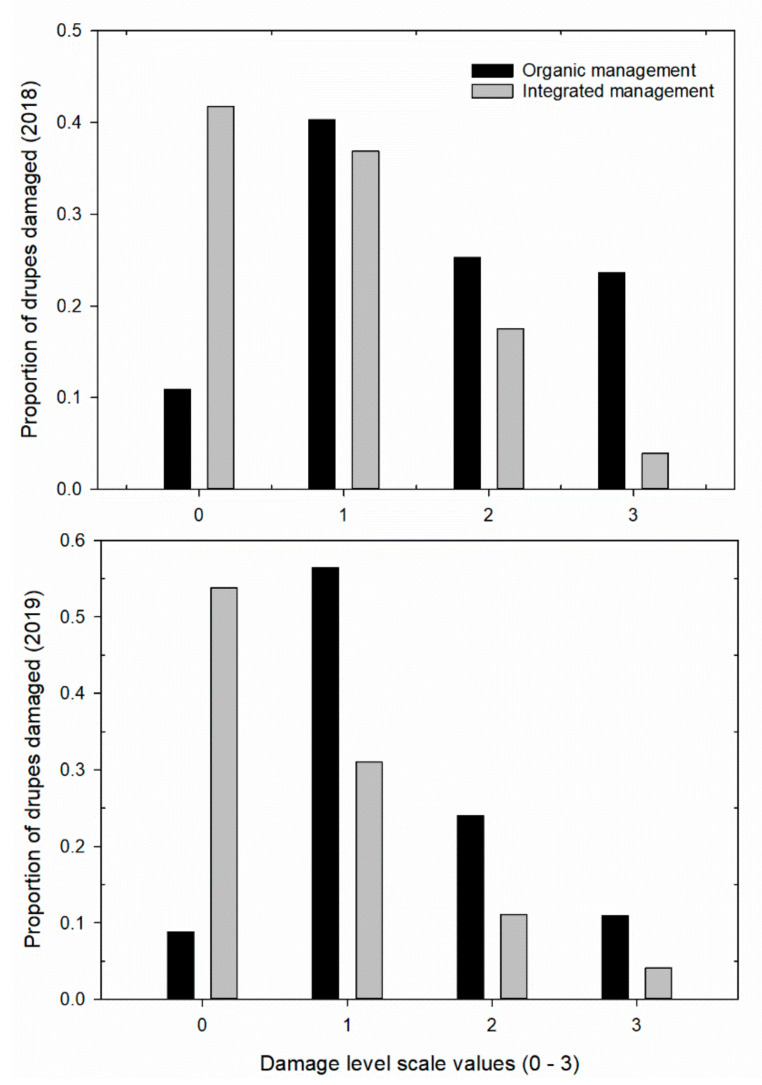
Proportion of drupes damaged by *Liothrips oleae* at different levels of damage with different types of phytosanitary management (see Figure 1c). Damage to drupes was evaluated using a scale of 0 to 3 according to the number of necrotized stings (see Material and Methods).

**Figure 5:**
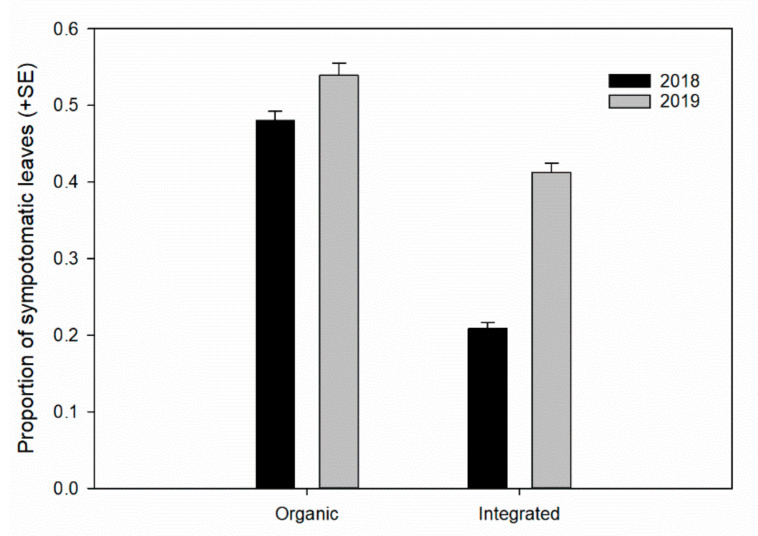
Proportion of leaves with symptoms of *Liothrips oleae* attack (see Figure 1b) with different types of phytosanitary management.

The generalized linear model analysis showed that the attack level was related to different variables; among these, phytosanitary management had an effect on the level of attack on the drupes and on the symptomatic leaves (Table 3). The organic drupes were more damaged by the thrips in all years studied (Figure 4). Moreover, the damage to the leaves was also higher with organic management (Table 3 and Figure 5). The average drupe diameter had an effect on the attack level of drupes, and, inversely, the exposure sector did not affect the level of damage to the drupes or the number of symptomatic leaves (Table 3).

Drupe size was negatively related to the attack level (2018: coefficient *B* = −3.295 (0.274), Wald Chi-square = 144.64, *p* < 0.001; 2019: coefficient *B* = −2.835 (0.266), Wald Chi-square = 113.22, *p* < 0.001, *n* = 1600). The average diameter of the drupes was lower in the organic management system of the olive grove in 2018 (organic drupe mean diameter = 1.509 (0.007) cm, *n* = 800; integrated mean diameter = 1.708 (0.006) cm, *n* = 800), while in 2019, the average diameter was similar (organic mean diameter = 1.614 (0.007) cm, *n* = 800; integrated drupe mean diameter = 1.545 (0.005) cm, *n* = 800). The comparison between the level of damage and symptomatic leaves produced a positive value (2018: *r* = 0.317; *p* < 0.001, df = 1597; 2019: *r* = 0.42; *p* < 0.001, *n* = 1597). There was significant variation in the premature drupes that dropped from the canopy according to different types of phytosanitary management and secondarily, with respect to direction of sun exposure (Table 4). The mean number of drupes that prematurely dropped on the soil due to thrips activity varied between the two types of orchard management and was significantly higher in the biologically managed orchard (mean: O = 12.38 ± 0.75 vs. I = 7.016 ± 0.39) and less in the NE sector of exposure (mean SE = 9.958 ± 0.653 > mean NE = 8.062 ± 0.957).

## 4. Discussion and Conclusions

The unexpected heavy infestations and the serious damage (necrosis and desiccation of sprouts and dropping of drupes) recorded in recent years have prompted researchers of thrips linked to Mediterranean agro-ecosystems to undertake monitoring activities of olive groves in order to evaluate the possible predisposing factors to the pest activity of this thrips species.

In olive ecosystems, as in other agrosystems [35,36], disrupting key predators has probably reduced effective suppression of other pests, as it may lead to secondary outbreaks. This hypothesis, however, was not confirmed from the data obtained from organic olive groves herein. In fact, the herbivore was particularly harmful to this type of management system. Some studies have shown that climate change can be considered the cause of alterations in the dynamics of populations of thrips species and other insects [37,38]. Ouyang et al. [39] suggested that climate change and agricultural intensification of the Anthropocene could potentially induce outbreaks of many pest insects by weakening the density-dependent population regulation. Other factors are to be considered, such as those related to particular microclimatic variations or sudden hot or cold snaps [40] that have probably affected the area and that is being analyzed with new research.

The results highlighted that the analyzed herbivore is capable of damaging the drupes and leaves throughout the crown of the plant, without revealing any preference regarding the exposure of the foliage (Table 3). It should also be pointed out that the number of drupes that dropped as a result of thrips’ sucking puncture was greater in organically managed olive groves. In order not to confuse the attacks on the drupes with those of other carpophages (e.g., *B. oleae*), a careful observation of drupes is necessary. However, the results of the prematurely dropped drupes confirmed that strong attacks on the foliage (leaves and fruits) are closely related to the dropping of drupes. The level of attack on drupes, and therefore, the number of punctures was negatively related to the average diameter of the drupes (Table 3 and estimated relative coefficient *B*). In fact, attacks on the drupes caused an arrest of their growth, highlighting how the studied herbivore is very harmful to the olive tree. Injury to various plant tissues by the feeding activity of thrips species on pollen, flowers, fruit, and leaves varies for different commercial crops, as does the range of damage and differences arising through attacks on the same plant at different stages of development [41]. The correlation between the levels of damage to the drupes and symptomatic leaves highlighted that these herbivores attack high-density symptomatic leaves and drupes with no preference.

In order to contribute to the correct identification of the species and to provide data that may constitute useful knowledge in the implementation of intervention protocols, the following contributions were made: The description of the species based on the morphological characteristics of the adults, their economic importance, the variation in their essential biology, and their relationships with olive-growing management. Olive crop production is hampered by pests that reduce the production and quality of olive oil [42], and this thrips species is re-emerging as a primary pest because its feeding on the drupes determines the suberification of the mesocarp, making the fruit unsuitable for olive milling and pickled olives. Although pest organism management information is available from different sources, their identification as *L. oleae* is difficult and often requires consultation with a specialist. DNA sequences generated by PCR have the potential to be extremely useful tools in the identification of pest species. The molecular characterization of *L. oleae* on three distinct genetic regions (i.e., *COI*, *ITS2*, and *28S*) was provided to support morphological identification. The sequencing of the PCR products showed no molecular differences among the olive thrips populations collected in the investigated areas. As investigated for other Phaleothripidae species, a short geographical distance would not be a determinant factor in the structure and genetic diversity of populations [43]. No molecular data on *L. oleae* were available in genetic databases before this study, which coupled morphological identifications with subsequent barcode analysis. The maximum likelihood phylogenetic tree based on mitochondrial gene revealed that *L. oleae* is well separated from all other *Liothrips* species (Figure 3). As recently described, Marullo et al. [44] also successfully distinguished some thrips species of the same genera based on *mt-*COI gene sequences. Furthermore, COI gene sequencing possibly identifies unknown specimens by comparing their COI sequence, and it has been used for identification purposes in projects known as species barcoding. Furthermore, as described by Inoue and Sakurai [45] and Buckman et al. [46], the amplification of mitochondrial and nuclear gene fragments (ITS2 and 28S) may prove useful in studies on the intra- and interspecific genetic variability of a species and its evolutionary relationships and phylogeny. Basically, biodiversity and polymorphism can be seen from DNA sequences of certain fragments of an organism genome [47].

No evidence relating to the containment of this pest was found in these two years of investigation. Further studies will aim to identify the causes of this resurgence of the pest and the containment factors of the species.

## Figures and Tables

**Table 1:** Locations and different management systems of olive groves used in the comparison of damage caused by *Liothrips oleae*. Organic management (O), integrated management (I).

Sites	Location	Province	Latitude	Longitude	Management
1	Stalettì	Catanzaro	38°45′45.9″ N	16°31′10.3″ E	O
2	Stalettì	Catanzaro	38°64′24.4″ N	16°32′7.2″ E	O
3	Belcastro	Catanzaro	38°59′35.7″ N	16°50′28.9″ E	I
4	Belcastro	Catanzaro	39°1′30.4″ N	16°48′34.2″ E	I

**Table 2:** Information about *Liothrips oleae* and the accession numbers related to the gene sequences of the samples analyzed.

Thrips Population Code	Date of Record	Host Plant	Location	Coordinates	Gene Sequences	Accession Number
1LO4	17/05/2018	*Olea europea* Carolea cv.	Stalettì (CZ)	38°45′45.9″ N 16°31′10.3″ E	COI	MT466525
					ITS2	MT559509
					28S	MT498786
MLO9	08/06/2018	*Olea europea* Carolea cv.	Belcastro (CZ)	38°59′35.7″ N 16°50′28.9″ E	COI	MT466530
					ITS2	MT559514
					28S	MT498796

**Table 3:** Generalized linear model (GLM) evaluating the main effects on the damage level of drupes (2018: *n* = 1600; 2019: *n* = 1600) and symptomatic leaves (Figure 1b,c). Organic management (O), integrated management (I).

Year	Drupes				Leaves			
	Level of Damage to Drupes	df	Wald Chi-Square	*p*	Symptomatic Leaves	df	Wald Chi-Square	*p*
**2018**	Management (O, I)	1	83.09	<0.001	Intercept	1	1879.67	<0.001
	Sectors of exposure	4	8,88	0.064	Management (O, I)	1	173.45	<0.001
	Average diameter drupe	1	144.64	<0.001	Sectors of exposure	4	9.25	0.055
**2019**	Management (O, I)	1	356.65	<0.001	Intercept	1	2056.03	<0.001
	Sectors of exposure	4	3.88	0.422	Management (O, I)	1	11.26	0.002
	Average diameter drupe	1	113.22	<0.001	Sectors of exposure	4	16.85	0.001

For the damage level, the likelihood ratio Chi-square was 427.37 (df = 6; *p* < 0.001) and 452.07 (df = 6; *p* < 0.001) for 2018 and 2019, respectively. For symptomatic leaves, the likelihood ratio Chi-square was 162.90 (df = 5; *p* < 0.001) and 27.94 (df = 5; *p* < 0.001) for 2018 and 2019, respectively. Sectors of exposure: northeast (NE), southeast (SE), southwest (SW), northwest (NW), and the middle of the plant.

**Table 4:** Generalized linear model (GLM) evaluating the effects of different variables on the dropped drupes (2018–2019: *n* = 192).

Source	df	Wald Chi-Square	*p*
Intercept	1	8288.32	<0.001
Year	1	0.88	0.065
Sectors of exposure	3	10.730	0.013
Management (O, I)	1	135.55	<0.001

Sectors of exposure (*n* = 4): NE, SE, SW, and NW.
